# Treatment response classes in major depressive disorder identified by model-based clustering and validated by clinical prediction models

**DOI:** 10.1038/s41398-019-0524-4

**Published:** 2019-08-05

**Authors:** Riya Paul, Till. F. M. Andlauer, Darina Czamara, David Hoehn, Susanne Lucae, Benno Pütz, Cathryn M. Lewis, Rudolf Uher, Bertram Müller-Myhsok, Marcus Ising, Philipp G. Sämann

**Affiliations:** 1 Max Planck Institute of Psychiatry, Munich, Germany; 2 Department of Neurology, Klinikum Rechts der Isar, School of Medicine, Technical University of Munich, Munich, Germany; 3 Social, Genetic and Developmental Psychiatry Centre, Institute of Psychiatry, Psychology, and Neuroscience, King’s College London, London, United Kingdom; 4 Department of Medical and Molecular Genetics, Faculty of Life Sciences and Medicine, King’s College London, London, United Kingdom; 5 Department of Pathology and Department of Psychiatry, Dalhousie University, Halifax, NS Canada; 6 Munich Cluster of Systems Biology, SyNergy, Germany; 7 Institute of Translational Medicine, University of Liverpool, Liverpool, UK

**Keywords:** Depression, Predictive markers

## Abstract

The identification of generalizable treatment response classes (TRC[s]) in major depressive disorder (MDD) would facilitate comparisons across studies and the development of treatment prediction algorithms. Here, we investigated whether such stable TRCs can be identified and predicted by clinical baseline items. We analyzed data from an observational MDD cohort (Munich Antidepressant Response Signature [MARS] study, *N* = 1017), treated individually by psychopharmacological and psychotherapeutic means, and a multicenter, partially randomized clinical/pharmacogenomic study (Genome-based Therapeutic Drugs for Depression [GENDEP], *N* = 809). Symptoms were evaluated up to week 16 (or discharge) in MARS and week 12 in GENDEP. Clustering was performed on 809 MARS patients (discovery sample) using a mixed model with the integrated completed likelihood criterion for the assessment of cluster stability, and validated through a distinct MARS validation sample and GENDEP. A random forest algorithm was used to identify prediction patterns based on 50 clinical baseline items. From the clustering of the MARS discovery sample, seven TRCs emerged ranging from fast and complete response (average 4.9 weeks until discharge, 94% remitted patients) to slow and incomplete response (10% remitted patients at week 16). These proved stable representations of treatment response dynamics in both the MARS and the GENDEP validation sample. TRCs were strongly associated with established response markers, particularly the rate of remitted patients at discharge. TRCs were predictable from clinical items, particularly personality items, life events, episode duration, and specific psychopathological features. Prediction accuracy improved significantly when cluster-derived slopes were modelled instead of individual slopes. In conclusion, model-based clustering identified distinct and clinically meaningful treatment response classes in MDD that proved robust with regard to capturing response profiles of differently designed studies. Response classes were predictable from clinical baseline characteristics. Conceptually, model-based clustering is translatable to any outcome measure and could advance the large-scale integration of studies on treatment efficacy or the neurobiology of treatment response.

## Introduction

Developing a major depressive disorder (MDD) and recovering from it is a dynamic process. While consensus definitions of MDD include core symptoms such as anhedonia and a depressed mood^1^, multiple additional symptoms may co-occur during an episode, each with individual patterns and variability throughout the episode^2,3^. During the development of a MDD, patients may go through sub-clinical phases with areas of preserved functioning in daily life, yet already show impaired psychosocial stress tolerance^4,5^. Strong inter-individual differences in the sensitivity towards psychosocial stress—a major risk factor for MDD^6^—may underlie such symptom plurality. Similarly, the regression of symptoms under treatment shows strong inter-individual differences. However, it is hypothesized that stable subgroups^7–9^ and predictive clinical patterns^8–13^ exist.

The latter is important for the successful clinical management of MDD. Treatment should ideally lead to full remission, as the persistence of residual symptoms increases the likelihood of a relapse^14^. Accordingly, delays of treatment intensifications or a switch of medication further increases the risk of treatment resistance and chronification^15^. Early treatment response (e.g., within 2 weeks) is particularly predictive of the longer course^16^—an established finding that also applies to outpatients and patients receiving a first-time antidepressant treatment^17^. Similarly, distinct psychopathological profiles reflect differences in the sensitivity of functional domains to stress and may be predictive of response patterns. For example, a patient suffering from severe anhedonia as a core symptom may respond particularly well to a treatment that addresses the dopaminergic system ^18^.

Due the heterogeneous symptomatology of depression, treatment response classes are typically based on compound scores on which relative change criteria or absolute thresholds are then applied (e.g., depression severity below a certain threshold over a defined time period). Different multivariate statistical techniques have been employed to identify predictive patterns for such conventional treatment response classes^10,12,13^. Chekroud et al.^10^ used an elastic net to identify 25 out of 164 patient-reportable variables of the Sequenced Treatment Alternatives to Relieve Depression (STAR*D) study that predicted response to citalopram. These variables were used to train a machine learning model, which could be validated with significant, yet low accuracy (59.7%) in an external sample. Nie et al.^12^, using data from the STAR*D study, trained five different machine learning algorithms on the full (700) or differently reduced (30 and 22, respectively) sets of clinical features to predict treatment resistance and non-resistance in STAR*D (at week 12) and an independent study (at week 6). Predictions were carried by early response markers and reached moderate accuracy. Wardenar et al.^13^ reported that the effect of information on comorbidities significantly improved the prediction of depression persistence and severity. Yet, while the here predicted response classes are mostly rooted in the long-known importance of early response and full remission, they are not data-based and may thus not represent all patterns contained in the data. Here, clustering analysis may be useful to dissect the dataspace into subspaces based on features that are shared within a subgroup and distinct between subgroups^19^. Clustering analysis has so far mainly been applied towards cross sectional markers to identify subgroups based on clinical symptom profiles^20–23^, cognitive markers^24^, or functional imaging markers^25^, assuming that clusters could indirectly reflect distinct pathophysiological components. Here, we attempt to cluster the treatment response space based on (total) symptom severity trajectories, i.e., the patients’ clinical development over a defined observation period. Longitudinal latent class analysis has reported five^26^ or nine such prototypical trajectories^27^ based on 12 weeks of observation. More specifically, the first study^26^ demonstrated rather limited prediction from ~13 clinical baseline items and polygenic scores formed from five literature-based, treatment associated genetic variants. The second study^27^ reported weak associations of response trajectories with the type of medication, yet investigated no clinical predictors. Another study revealed seven trajectories based on 1 year of observation^28^, yet, no prediction models were tested. One limitation of these studies, however, is their narrow generalizability as data from single centers studies were used.

In order to understand whether treatment response classes (TRC[s]) are specific to a study site-specific patient selection and treatment approach or whether they represent a generalizable dynamical fingerprint, we included two types of cohort studies in our work: First, the Munich Antidepressant Response Signature (MARS) study, a prospective, open, observational trial performed at the MPI of Psychiatry, Munich, and collaborating hospitals^29^. Second, the Genome-based Therapeutic Drugs for Depression (GENDEP) study, a partially randomized, multicenter clinical and pharmacogenomic study^30^. In both studies, the Hamilton Depression Rating Scale (HAM-D), which achieves good test-retest and interrater reliabilities^31^ was used to assess current symptom levels, covering most domains that define MDD such as depressed mood, suicidality, anhedonia, lack of drive, circadian symptom changes, and autonomous nervous system disturbances.

The aims of this study were (i) to establish TRCs in a data-driven fashion, based on serial depression ratings as acquired during studies with naturalistic or partially randomized treatment, and (ii) to assess the generalizability and clinical validity of the resulting TRCs. For this purpose, we applied a mixed model-based, non-linear longitudinal clustering technique to detect TRCs (also referred to as clusters) in MDD in our discovery sample, a subsample of the MARS cohort. The core feature of this clustering technique is to assigns individuals to a cluster (here: a TRC) by while borrowing information from all other individuals and hereby improving cluster stability, which often is critical for generalizability and clinical applications. For the second aim, we assessed cluster generalizability empirically in a second subsample of MARS (MARS validation sample) and in the GENDEP sample, and employed random forest analyses to explore if clinical characteristics at baseline can predict the TRCs in the MARS discovery and validation sample.

## Methods and materials

### General study samples characterization

Both the MARS and the GENDEP study protocol were approved by the respective local ethics committees. All participants gave their written informed consent before participation. MARS patients were admitted to the hospital of the MPIP, Munich, Germany, or collaborating hospitals in southern Bavaria and Switzerland for the treatment of different depressive disorders. Started in 2000, the study aimed at generating a large database of longitudinal observations with weekly ratings along with sociodemographic, psychopathological, and biological data from in-patients with all types of depressive disorders including MDD, bipolar depression, and schizoaffective disorder^29^. Diagnoses according to ICD10^32^ were obtained from trained psychiatrists using patient interviews and clinical documentation^29^. Of 1286 available patients, only patients with either a single episode of MDD (ICD-10 F32, *N* = 373) or a recurrent (unipolar) depressive episode (ICD-10 F33, *N* = 698) were eligible. Patients with bipolar depression (*N* = 175), chronic depression (ICD-10 F34, *N* = 3), or patients with a baseline HAM-D score <14 (*N* = 37) were excluded. Of the remaining 1071 datasets suitable for this study, 834 patients (recruited 2002–2011) formed the discovery sample and 236 patients (recruited 2012–2016) the MARS validation sample. The split point represented an organizational intercept related to genotyping activity unrelated to this study. The age range was 18–87 years (see Table 1 for demographic and clinical details). Patients were treated psychopharmacologically according to the attending doctor’s choice and received therapeutic drug monitoring to optimize plasma medication levels. Depression symptoms were evaluated weekly using the 21-item version of the HAM-D until week 6 and, after that, bi-weekly until discharge or, if not discharged, until week 16 as the latest assessment. During the first six weeks, 7.1% of the HAM-D scores were accidentally missing due to organizational reasons. Accidentally missing HAM-D scores of the first 6 weeks and bi-weekly skipped HAM-D scores between week 6 and 16 were linearly interpolated from the previous and subsequent scores to obtain complete time series. Eighty-eight percent of patients of the discovery and 99% of the MARS validation samples were discharged before week 16 and thus provided HAM-D time series with fewer than 17 data points.

**Table 1:** Description of clinical items used for multivariate prediction models in the MARS cohort

Category	Model	Variable description	Short name	Type	MARS discovery sample		MARS validation sample		*p*-value^a^
					Mean	SD	Mean	SD	
					%		%		
Sociodemographic data	0	Age at study inclusion (years)	age	*N*	48.26	14.02	45.48	14.99	0.008^c^
	0	Sex (% female)	sex	*D*	53.72 %		53.39 %		0.941
	0	Living with a partner	spouse	*D*	50.24%		38.56%		0.001^b^
	0	School years of education (university not considered) (years)	education	*N*	10.33	1.46	10.21	1.51	0.275
	0	Being in training/retirement vs. employment	training_retirement	*D*	25.42%		22.46%		0.393
	0	Employment status: unemployed/part time/full time	employment	*N*	1.54	0.78	1.56	0.78	0.678
Diagnosis	0	ICD-10 code for recurrent depressive disorders (F33) (%)	ICD10	*C*	64.63%		66.95%		0.536
History of depressive disorder	0	Age at disease onset (years)	age_on	*N*	36.51	15.16	34.06	14.26	0.027^b^
	0	Number of previous depressive episodes	prev_epi	*N*	2.62	5.24	2.58	3.69	0.894
	0	Any suicide attempt before current episode	s_history	*D*	19.54%		9.32%		0.0001^c^
	0	Psychotic symptoms in any previous episode	psychot_history	*D*	11.15%		3.81%		0.0004^c^
Family history	0	Family history of any mental disorders	fam_history	*D*	63.19%		64.41%		0.760
	0	Family history of schizophrenic disorders	fam_F20_F25	*N* ^d^	0.08	0.37	0.06	0.32	0.340
	0	Family history of bipolar disorders	fam_F31	*N* ^d^	0.05	0.31	0.08	0.37	0.348
	0	Family history of affective disorders (except bipolar disorder)	fam_F32_ _F34	*N* ^d^	0.88	0.95	0.87	0.96	0.857
	0	Family history of attempted suicide	fam_X60	*N* ^d^	0.23	0.58	0.16	0.46	0.082
Information on current episode	0	Duration of the current episode (weeks)	index_d	*N*	34.54	58.74	32.19	51.58	0.577
	0	ATRQ total score of treatment resistance for pre-medication	ATRQ_score	*N*	1.09	0.90	1.01	1.33	0.311
	0	Suicide attempt during the current episode	s_current	*D*	10.31%		2.54%		<0.0001^c^
	0	Psychotic symptoms during the current episode	psychot_current	*D*	10.43%		2.97%		0.0001^c^
Basic medical and baseline laboratory data	0	Body height (m)	height	*N*	1.72	0.09	1.72	0.09	0.545
	0	Body weight (kg)	weight	*N*	25.65	6.07	25.94	5.28	0.504
	0	Body mass index (m 2/kg)	BMI	*N*	25.34	4.41	25.94	5.27	0.075
	0	Heart rate (1/min)	HR	*N*	82.75	13.16	80.45	12.14	0.016^b^
	0	Systolic blood pressure (mm Hg)	RRsys	*N*	125.78	18.10	128.04	17.44	0.088
	0	Diastolic blood pressure (mm Hg)	RRdia	*N*	78.70	11.06	79.10	11.97	0.640
	0	Morning cortisol level (µg/l)	cort_basal	*N*	200.53	39.61	206.70	63.54	0.068
	0	Thyroid stimulating hormone level (µIU/l)	TSH	*N*	1.47	1.02	1.75	1.21	0.0005^c^
	0	Free T3 hormone level (pmol/l)	fT3	*N*	4.57	0.93	4.45	0.62	0.065
	0	Free T4 hormone level (pmol/l)	fT4	*N*	16.16	9.23	15.29	3.57	0.158
	0	CRP level (mg/l)	CRP	*N*	1.49	2.92	2.83	9.00	0.0002^c^
	0	HBA1c level (mmol/mol)	HbA1C	*N*	5.34	0.34	5.31	0.35	0.209
Life events	0	Sum of life events	L-Event	*N*	29.50	10.46	30.23	11.83	0.359
	0	Stress-weighted sum of life events	wL-Event	*N*	82.30	38.65	86.36	47.94	0.177
Baseline psychopathology	0	Symptom checklist-90-R (SCL-90R) for somatization	scl_som	*N*	0.97	0.64	0.99	0.64	0.488
	0	SCL-90R for compulsiveness	scl_comp	*N*	1.77	0.72	1.70	0.69	0.177
	0	SCL-90R for uncertainty in social contact	scl_uncert	*N*	1.30	0.77	1.33	0.83	0.630
	0	SCL-90R for depression	scl_dep	*N*	2.08	0.73	2.06	0.76	0.660
	0	SCL-90R R for anxiety	scl_anx	*N*	1.37	0.70	1.31	0.75	0.258
	0	SCL-90R for aggressiveness/hostility	scl_agg	*N*	0.77	0.60	0.86	0.69	0.046^b^
	0	SCL-90R for phobic anxiety	scl_pho	*N*	0.88	0.75	0.94	0.83	0.283
	0	SCL-90R for paranoid ideation	scl_par	*N*	0.92	0.72	0.99	0.82	0.218
	0	SCL-90R for psychoticism	scl_psy	*N*	0.83	0.55	0.80	0.54	0.507
Personality items	0	Eysenck Personality Questionnaire (EPQ)-RK neuroticism	epq_neu	*N*	6.85	2.50	6.84	2.73	0.938
	0	EPQ-RK psychoticism	epq_psy	*N*	1.92	1.24	2.16	1.40	0.010^b^
	0	EPQ-RK extraversion	epq_ext	*N*	5.20	2.97	5.07	3.03	0.567
	0	Tridimensional Personality Questionnaire (TPQ) Harm avoidance total	tpq_ha	*N*	20.63	5.58	20.27	5.92	0.386
	0	TPQ Novelty Seeking total	tpq_ns	*N*	13.07	3.81	14.04	4.41	0.001^b^
	0	TPQ Reward Dependence total	tpq_rd	*N*	17.75	3.30	17.50	3.84	0.318
	0	TPQ Reward Dependence–Subscale Persistence	tpq_rd2	*N*	4.81	1.70	4.88	1.86	0.623
HAM-D single items (baseline)	1, 3	21 HAM-D single items (baseline)	HAM-D0_01-HAM-D0_21	*N*	N/T^e^	N/T^e^	N/T^e^	N/T^e^	N/T^e^
Early partial response (at week 2)	2, 3	HAM-D early partial response (≥25% reduction) after 2 weeks	HD_2WE	*D*	N/T^e^	N/T^e^	N/T^e^	N/T^e^	N/T^e^

*N* numerical, *D* dichotomous, *C* categorical

^a^Two-sided comparisons between the MARS discovery and validation samples (Fisher’s exact test and Fisher-Freeman-Halton test for dichotomous and categorical variables; Student’s *t*-test for numerical variables)

^b^Nominal significance (*p* < 0.05)

^c^Significan^c^e after Bonferroni correction for multiple testing, here: *p* < 0.05/50 = 0.001

^d^To allow optimal use in a parametric test, variables were coded as 0 (no relative affected), 1 (only second-degree relatives affected), and 2 (first-degree or first-degree and second-degree relatives affected).

^e^Not tested as these it^e^ms were not part of model 0.

The GENDEP study represents a partially randomized, multicenter clinical, and pharmacogenomic study on depression^33^ into which 826 subjects were enrolled between July 2004 and December 2007. The main inclusion criterion was the diagnosis of a major depressive episode of at least moderate severity as defined by DSM-V^1^ and ICD-10 criteria^32^ and as established by the Schedules for Clinical Assessment in Neuropsychiatry (SCAN, version 2.1)^34^. Exclusion criteria were a first-degree relative with bipolar affective disorder or schizophrenia, a history of a hypomanic or manic episode, mood incongruent psychotic symptoms, primary substance misuse, primary organic disease, current treatment with an antipsychotic or a mood stabilizer, and pregnancy or lactation. Patients eligible for both antidepressants were randomly allocated to receive either nortriptyline (50–150 mg/day) or escitalopram (10–30 mg/day) for 12 weeks with clinically informed dose titration. Patients with a history of adverse effects, non-response or contraindications to one of these drugs were non-randomly allocated to the other drug. Patients who could not tolerate the initially allocated medication or who did not experience sufficient improvement with adequate dosage within 8 weeks were offered the other antidepressant. Depression symptoms were evaluated weekly until week 12 by psychiatrists or psychologists using the 17-item version of the HAM-D score^35^. The age range of all subjects was between 18 and 72 years, all patients were of European ethnicity. A total of 15 subjects who had missing data on all three suicidality items at baseline were excluded, as were patients with a baseline HAM-D score <14, leaving 809 patients for analysis^35^. Demographic data are given in Supplementary Table S1. Different biological aspects of treatment response^36,37^ and psychopathological predictor schemes have been reported from this study ^27^.

### Clustering algorithm

A mixed model approach was used to describe the course of the individual HAM-D score time series, after (natural-) logarithm (ln) transformation (ln of [HAM-D scores +0.5]), considering information not only from the individual trajectory, but combining trajectories of several patients to identify TRCs. For a first organization of HAM-D responses into TRCs, we applied the *FlexMix*^38,39^ clustering algorithm in *R* (version 3.3) on the HAM-D trajectories of the MARS discovery sample. *FlexMix* provides an infrastructure for the flexible fitting of finite mixture models using the expectation-maximization algorithm to cluster individual trajectories. The algorithm iterates between computing the expectation of the log-likelihood and maximizing it to find the parameters of the TRCs. To achieve a stable cluster solution, we ran the clustering model with 200 repetitions and determined the optimal number of TRCs based on the Integrated Completed Likelihood (ICL) criterion generated by the model.

To validate the stability and generalizability of the clustering solution, the coefficients of the model of the discovery sample were projected onto a second, later acquired subsample of the same cohort, referred to as MARS validation sample (*N* = 236). Here, the hypothesis was that the patients are classifiable into the defined TRCs with approximately equal proportions and similar cluster-wise median HAM-D courses as had been observed for the discovery sample. In addition, we projected the same clustering model onto 12-week HAM-D courses of the GENDEP sample, hypothesizing similar median HAM-D courses per class, yet, not necessarily similar cluster proportions due to differences in the patient population and the study design. For both projection experiments, resulting proportions of classes were compared with the original distribution of the discovery sample using a χ^2^ test. In order to assess suitability of the clustering solution for the validation samples, posterior likelihood values, classification log-likelihoods and eventually ICL values for were calculated on the basis of the clustering model of the discovery sample.

To assess the applicability of the original clustering coefficients to samples with a shorter observation interval, we systematically lowered the number of applied coefficients down to 1 and, for each observation interval report, compared this classification with the classification based on all coefficients (i.e., the full observation interval). The true distance (or dis-correlation) between the two solutions was calculated by Pearson correlation between model-based slope values of the respective TRC.

### Multivariate prediction analyses

We then conducted a multivariate analysis using a random forest algorithm as implemented in the *R* package *Ranger*^40^ to detect associations between clinical variables and the previously obtained TRCs in the MARS sample.

#### Clinical predictors

All 72 clinical variables are explained in Table 1. Their selection was based on two rationales: First, availability in both MARS subsamples and, second, preference of such variables that are based on broadly available measurement instruments. The main model (model 0) comprised 50 clinical variables strictly from the baseline assessment, covering the domains of sociodemographic data, clinical diagnosis, history of the MDD, the current episode, psychiatric family history, basic laboratory data, life events, the current psychopathology (Symptom Checklist [SCL-90R])^41^, and personality questionnaires (Eysenck Personality Questionnaire [EPQ]^42^, Tridimensional Personality Questionnaire [TPQ]^43^). As random forest models require complete datasets, missing data were filled by the respective median of the total sample (for details see Supplementary Table S2). Extended models were: Model 1, which is model 0 expanded by 21 baseline HAM-D single items to investigate the effect of unfolding the baseline psychopathology; model 2, which is model 0 expanded by the partial response at week 2 to investigate the influence of early longitudinal observations; model 3, the combination of both expansions (Supplementary Fig. S1).

#### Random forest-based prediction models

The basic algorithm used in the *Ranger* package is a fast implementation of random forests for high dimensional data. In a random forest, each node is split using the best among a subset of predictors randomly chosen at that node^44^. Two parameters were used to control this process: the number of prediction trees (*bagging*) and the number of features to search across to find the best feature (*mtry*). *Mtry* is the square root of *D*, which is the number of independent predictors used for classification. Predictions were obtained by aggregating the prediction trees (i.e., the majority votes for classification and the average for regression models). We calculated adjusted coefficients of multiple correlation *R*^2^ (to quantify the explained variance and predictive quality of the entire model) and corresponding *p*-values. To characterize feature importance, a permutation based method that exploits the distribution of measured importance for each variable in a non-informative setting was applied^45^ (10000 permutations); predictors with *p* < 0.05 are reported in more detail. Further, differences in *R*^2^ between competing models were compared after Fisher’s Z-transformation of the respective *r* values.

Prediction models were estimated on the pooled discovery and validation MARS sample. For each set of predictors, two ways of modeling the HAM-D time series were considered: first, the patient’s individual treatment response slope, a simple linear regression on ln-transformed HAM-D values, and, second, the slope derived from the clustering model. The rationale for this comparison was to determine the quality of the clustering method to generate meaningful and generalizable outcome classes. Further, class specific classification accuracy values (i.e., [true positives + true negatives]/[true positives + false positives + true negatives + false negatives]), were calculated on the basis of respective confusion matrices in which the class of interest was defined as true class, and the remaining six other classes as false class.

## Results

### Clustering of HAM-D time courses

**Fig. 1:**
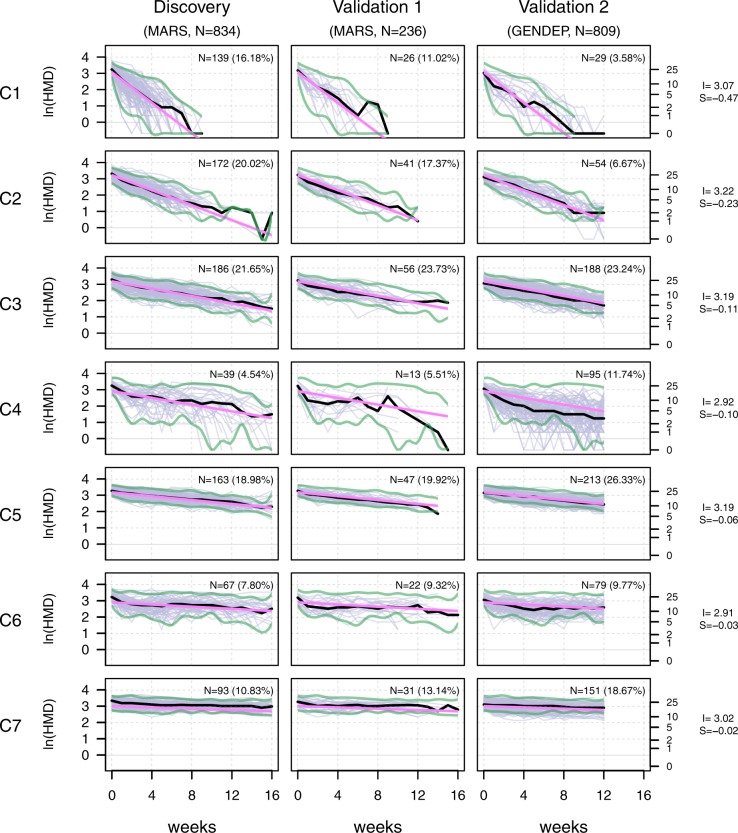
Resulting cluster shape characteristics and underlying natural logarithm-transformed HAM-D courses for the discovery sample and both validation samples. *X-axis:* observation time in weeks; *Y-axis:* natural logarithm-transformed HAM-D values (purple: raw values, black: cluster-specific median, pink: model-based linear fit). Slope and intercept values of all clusters are given on the right. Clusters are sorted from C1 to C7 according to the cluster-specific slope. Absolute and relative cluster sizes in all samples are given within the subplots. Green borders represent the limits in which 95% of HAM-D values of the discovery sample were contained. These were transferred to columns 2 and 3 to allow for comparison with the validation samples. S slope, I intercept, ln natural logarithm-transformed

When applied to the HAM-D time courses of the discovery sample, the *FlexMix* clustering algorithm did not converge for any number of clusters k < 4 or k > 10. We therefore assessed cluster stability in more detail for k ≥ 4 and k ≤ 10, using 200 repetitions of the algorithm for each k. The lowest value of the ICL criterion, representing an optimal model fit, was found for seven clusters (Supplementary Fig. S2A). Figure 1 shows the resulting TRCs (C1 to C7), sorted by their model-derived slope. C1 showed the fastest symptom improvement, whereas C2 and C3 were characterized by improvements at slower rates. Cluster C4 reflected a more volatile symptom development, while C5, C6, and C7 were characterized by low improvement rates, with C7 showing practically no improvement over at least 16 weeks. Mean baseline HAM-D scores differed slightly between clusters (ANOVA, *p* = 0.009); mean average HAM-D scores of the episode differed strongly (ANOVA, *p* = 4.022 × 10^−116^). Cluster-derived slopes correlated weakly with baseline HAM-D (*r* = 0.09, *p* = 0.002) and strongly with average HAM-D scores of the episode (*r* = 0.57, *p* = 8.270 × 10^−76^) (Supplementary Table S3).

To examine whether the TRCs represent stable and generalizable entities, we assigned the patients of the two MARS- and GENDEP-based validation samples to clusters, using the coefficients of the model estimated in the discovery sample. Figure 1 compares the individual trajectories across the three samples and shows the respective cluster-specific median time courses along with boundaries that include 95% of values of the discovery sample. Supplementary Fig. S2B shows ICL values for both validation samples, separately and combined. All samples showed an ICL minimum for seven clusters except for the MARS validation sample. The latter showed a flat ICL profile with a relative minimum at five clusters, most likely due to the relatively small sample size of about 30% compared with the MARS discovery and the GENDEP validation sample. For the MARS validation sample we observed that median HAM-D courses were highly similar to the discovery sample and cluster proportions were not different (*Χ*^*2*^ = 6.157, *p* = 0.40). The GENDEP validation sample exhibited very similar median HAM-D courses compared with the discovery sample, except for C4, which had lower median values compared with MARS, caused by several patients with high volatility between week 4 and ~10 and HAM-D values below the 95% threshold. Compared with the MARS discovery sample, GENDEP clusters had different proportions (*Χ*^*2*^ = 177.13, *p* = 1.38 × 10^−35^), showing fewer fast responders (e.g., in C1, average 4.9 weeks to discharge) and more slow responders (e.g., in C7 average 20.8 weeks to discharge).

**Fig. 2:**
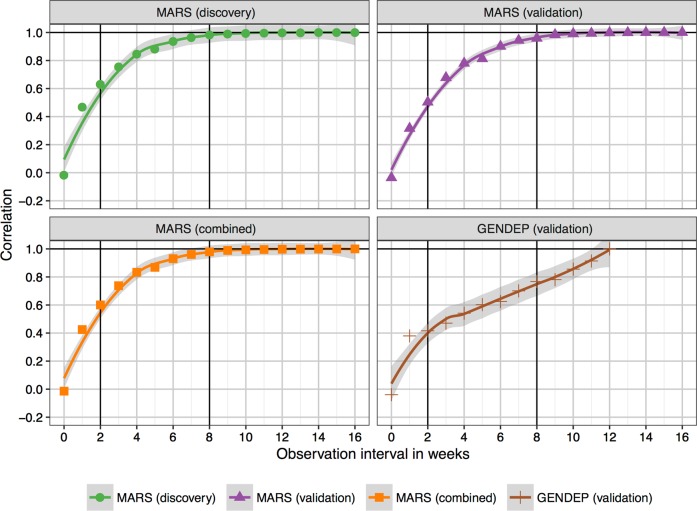
Prediction accuracy for reduced observation intervals. Correlation of prediction result achieved from reduced observation intervals ranging from one observation (baseline HAM-D) to the full set of either 17 HAM-D values (baseline through week 16, for MARS derived samples) or 13 HAM-D values (baseline through week 12, for GENDEP sample). Pearson correlations were calculated between clusters predicted using the reduced and predicted with the full observation interval, using the model-based HAM-D slope of the respective cluster. Note that a positive linear correlation of ≈0.50 was reached at week 2 and a correlation of ≈0.96 (for the MARS samples) and ≈0.77 (for GENDEP) was reached at week 8

Next, we analyzed to what degree a lower number of sequential observations would suffice to predict the TRCs instead of using the full observation interval. Here, we detected an almost linear increase of the correlation coefficient between the reduced and full solutions from week 0–4. Correlations were already high at week 8 for the MARS validation and the combined MARS sample (0.96–0.98) (Fig. 2). For GENDEP, as fully independent sample, the slope was generally lower, reaching 0.77 at week 8 and remaining linear until its maximum.

Strong correlations between the TRCs and established response markers (weeks until discharge, response [50% relative symptom decrease at discharge] and remission [HAM-D < 10 at discharge]) were confirmed (Supplementary Table S4). These differences were significant between ~80% of neighboring clusters, particularly for remission as a conservative criterion (Supplementary Table S5), highlighting an ecological importance of the cluster differences. Clusters also differed regarding the psychopharmacological treatment administered throughout the episode for three of nine medication classes (benzodiazepines, tricyclic antidepressant, and antipsychotics) (Supplementary Table S4).

### Predicting TRCs from clinical characteristics

We assessed whether the attribution of patients to the TRCs can be predicted from clinical characteristics. While explorative, the analysis served mainly as general cluster validation step by probing if the TRCs associate with clinically plausible and previously reported prediction patterns. To this end, we analyzed four models with a focus on model 0 that comprised 50 clinical baseline items. Model 1 comprised additional baseline HAM-D single items, model 2 contained the early partial response at week 2, and model 3 combined models 1 and 2. All four models predicted treatment response in the combined MARS sample for both alternatives of modelling the slope (individual and cluster-derived) (both *p* < 2.17 × 10^−21^, Table 2). Overall, two performance levels (A and B) were observed for models using the cluster-derived slope: (A) Model 0 and 1 both explained 13% of the variance, which means that no gain was achieved by inclusion of the baseline HAM-D single items. (B) Model 2 and 3 explained 20% and 21% of the variance, respectively, with the improvement over (A) induced by the early partial response item; as observed in the first comparison (A), no added effect of the baseline HAM-D single items was seen for model 3. Predictions were also significant for all four models when analyzing the two MARS subsamples (*p* < 1.30 × 10^−17^ and *p* < 8.71 × 10^−5^ for the discovery and validation sample, respectively). It is worth mentioning that for the MARS validation sample the prediction analysis was entirely independent from the clustering procedure. Across all models, using the cluster-derived slope explained significantly more of the variance than using the individual slopes (Table 2). Classification accuracies as calculated from cluster-specific confusion matrices ranged between 75.0% and 95.2% (Supplementary Table S6 for details).

**Table 2:** Prediction characteristics of model 0 and the extended models 1–3

Model	Sample	Explained variance (Adjusted *R*^2^)^a^		Overall model significance		Significance of the *R*^2^ difference (*p*-value)^b^
		Individual	Cluster-derived	Individual	Cluster-derived	
Model 0	All	0.08	0.13	2.17 × 10^−21^	1.53 × 10^−33^	0.019
Model 0	Discovery	0.08	0.12	3.76 × 10^−18^	1.54 × 10^−24^	0.106
Model 0	Validation	0.06	0.19	8.71 × 10^−5^	1.72 × 10^−12^	0.009
Model 1	All	0.08	0.13	4.35 × 10^−22^	1.49 × 10^−34^	0.025
Model 1	Discovery	0.08	0.12	1.30 × 10^−17^	2.06 × 10^−24^	0.097
Model 1	Validation	0.10	0.20	7.35 × 10^−7^	4.09 × 10^−14^	0.047
Model 2	All	0.13	0.20	1.52 × 10^−34^	3.42 × 10^−54^	0.008
Model 2	Discovery	0.14	0.21	6.78 × 10^−30^	8.43 × 10^−45^	0.026
Model 2	Validation	0.07	0.20	3.64 × 10^−5^	8.68 × 10^−14^	0.008
Model 3	All	0.13	0.21	2.95 × 10^−34^	1.53 × 10^−57^	0.004
Model 3	Discovery	0.13	0.21	2.42 × 10^−28^	1.71 × 10^−46^	0.012
Model 3	Validation	0.11	0.21	2.76 × 10^−7^	9.93 × 10^−15^	0.050

^a^Adjusted *R*^2^ coefficients indicate the explained variance and *p-*values indicate the overall model significance.

^b^Based on Fisher’s Z’-transformed *r* values

Table 3 lists 10 (out of 50) predictors of model 0 that gained significance based on a multivariate comparison of the respective single item against all other competing items^45^. We also analyzed univariate associations of these items with the TRCs (likelihood ratio test on a generalized linear model). Concordantly, both types of comparison revealed strongest effects for the personality items *neuroticism*, *extraversion*, and *harm avoidance*. Furthermore, we investigated the cluster-specific averages of each clinical item, comparing them to the 95% confidence interval (CI) of the entire sample (Table 3): Clusters with fast improvement (C1 and C2) showed below-average values of all predictors except for the personality trait of *extraversion*. By contrast, the treatment resistance cluster C7 showed above-average values of all items except for the personality items *extraversion* and *psychoticism*. More generally, except for *extraversion*, there was a tendency that lower clinical scores (i.e., a shorter *duration of the current episode*, less *SCL-90R symptoms*, fewer *stress-weighted life events*, and lower scores for the personality items *neuroticism* and *harm avoidance*) were found in clusters with good treatment response, and higher scores in clusters C6 or C7. Deviations from this pattern, mostly in the intermediate clusters C3–C5 (see, for example, the *stress-weighted life events*) may point towards non-linear relationships or complex interactions. No demographic variables were selected by the random forest algorithm. Still, to not overlook demographic variables that could have driven the clustering, we compared these between the clusters, particularly of the MARS discovery sample, finding no relevant differences (Supplementary Table S7).

**Table 3:** Univariate comparison of significant predictors between TRCs (model 0, combined MARS samples)

Cluster	Clinical items^a^																	
	index_d		scl_uncert		scl_psy		scl_pho		epq_neu		epq_ext		epq_psy		tpq_ha		wL-Event	
C1	22.27 ± 27.67	↓	1.04 ± 0.70	↓	0.63 ± 0.48	↓	0.64 ± 0.69	↓	5.17 ± 2.81	↓	6.42 ± 3.34	↑	1.91 ± 1.63	0	17.18 ± 6.23	↓	69.82 ± 33.76	↓
C2	28.46 ± 54.70	↓	1.19 ± 0.70	↓	0.74 ± 0.46	↓	0.79 ± 0.65	↓	6.59 ± 2.52	↓	5.65 ± 3.04	↑	1.94 ± 1.13	0	19.81 ± 5.65	↓	77.92 ± 29.51	↓
C3	43.35 ± 75.64	↑	1.41 ± 0.83	↑	0.84 ± 0.59	0	0.94 ± 0.79	0	7.43 ± 2.42	↑	4.95 ± 3.06	↓	2.00 ± 1.31	0	21.51 ± 4.86	↑	87.15 ± 44.22	↑
C4	17.23 ± 14.19	↓	1.02 ± 0.86	↓	0.67 ± 0.52	↓	0.63 ± 0.71	↓	5.92 ± 2.81	↓	5.56 ± 3.08	↓	2.35 ± 1.44	↑	18.15 ± 6.55	↓	29.00 ± 12.94	↓
C5	38.67 ± 60.82	↑	1.48 ± 0.76	↑	0.93 ± 0.50	↑	1.02 ± 0.75	↑	7.47 ± 2.19	↑	4.52 ± 2.39	↓	1.94 ± 1.10	0	21.92 ± 5.05	↑	86.46 ± 39.29	↑
C6	30.97 ± 34.81	0	1.27 ± 0.74	0	0.86 ± 0.58	↑	0.94 ± 0.84	0	7.00 ± 2.05	0	4.76 ± 2.88	↓	2.10 ± 1.27	↑	21.29 ± 4.73	↑	90.72 ± 45.12	↑
C7	43.46 ± 63.86	↑	1.53 ± 0.79	↑	1.03 ± 0.63	↑	1.18 ± 0.86	↑	7.74 ± 2.02	↑	4.26 ± 2.39	↓	1.87 ± 1.12	↓	22.81 ± 4.90	↑	94.96 ± 50.82	↑
95% CI^b^	30.59; 37.46		1.26; 1.35		0.79; 0.85		0.85; 0.94		6.70; 7.00		4.99; 5.35		1.90; 2.05		20.21; 20.89		80.74;85.65	
Multivariate importance *p-*value	0.0210		0.0073		0.0208		0.0182		<0.0001		<0.0001		0.0445		<0.0001		0.0002	
ANOVA *p-*value (Cohen’s *f* ^c^)	4.0 × 10^−4^ (0.153)		2.5 × 10^−10^ (0.233)		1.9 × 10^−10^ (0.234)		9.8 × 10^−10^ (0.227)		7.1 × 10^−25^ (0.355)		2.9 × 10^−11^ (0.243)		3.3 × 10^−1^ (0.081)		6.7 × 10^−23^ (0.341)		4.4 × 10^−7^ (0.196)	

^a^index_d: duration of the current episode; scl_uncert: uncertainty in social contact (SCL-90R); scl_psy: psychotism (SCL-90R); scl_pho: phobic anxiety (SCL-90R), epq_neu: neuroticism (EPQ-RK), epq_ext: extraversion (EPQ-RK), epq_psy: psychoticism (EPQ-RK), tpq_ha: harm avoidance total (TPQ), wL-Event: stress-weighted sum of life events. See Table 1 for more details on the clinical items.

^b^CI: confidence interval. Arrows indicate lower (↓), higher (↑), or within (0) positioning regarding the 95% CI of the respective parameter distribution.

^c^Cohen’s *f*: >0.10 and <0.25: small effect; ≥0.25 and <0.40, medium effect; ≥0.40: large effect.

Supplementary Table S8 summarizes significant predictors of the three extended models. In brief, model 1, compared to model 0, was characterized by prioritizing three *baseline HAM-D single items*; model 2 identified, as expected, *early partial response* as a strong predictor, along with minor other shifts; model 3 produced a combined pattern with *baseline HAM-D single items*, *early partial response*, and *current psychotic symptoms* as additional predictors over model 0.

## Discussion

We employed model-based non-linear clustering^38,39^ on symptom courses of 834 in-patients treated for MDD and identified seven TRCs. These classes were already distinct at the visual level and ranged from fast, unambiguous response to severe treatment resistance. The average HAM-D decrease differed strongly between classes, and classes were strongly associated with established response markers, highlighting that they represent clinically meaningful entities. Baseline severity was only weakly correlated with the response slope over a small HAM-D range, contradicting the intuitive expectation that a high initial disease severity is closely coupled to a steep symptom decline. Classification of 236 patients of the MARS validation sample and 809 patients of the GENDEP validation sample demonstrated that the patients’ response dynamics can be captured by these clusters, yet study-specific differences in the response profiles are also reflected.

### Construct validity of the clustering solution

Similar cluster sizes and shape characteristics emerged when the discovery sample coefficients were applied to the validation samples (Fig. 1). The consistency observed in this validation is superior to previous latent variables analyses not using machine learning, which did not produce stable, symptom-based subtypes of depression^3^. Still, a major difference that limits the comparability is that the mentioned analyses (factor analyses, principal component analyses, latent class analyses) built their grouping on cross sectional symptom spectrum and not on trajectories of symptom changes.

Here, we applied a machine learning strategy to identify MDD subtypes based on longitudinal data collected over up to 16 weeks. Our results indicate that significant latent subtypes for MDD indeed exist in the MARS cohort. One advantage of our approach may have been the identification of the best model through the ICL criterion that appears more robust to the violation of some of the mixture model assumptions compared with the commonly used Bayesian Information Criterion. Therefore, the use of the ICL may have led to a more optimal choice for the number of clusters and, accordingly, to a more sensible data partitioning ^46,47^.

Within each model, the use of slopes derived from the linear mixed model characterizing each TRC led to higher *R*^2^ coefficients than the use of individual slopes, particularly in the validation sample (Table 2). This observation strengthens the validity of the classes and highlights that the individual information of the HAM-D time courses was indeed assessed by the clustering algorithm. Moreover, this emphasizes that the average slope of the class is a good approximation of the response behavior, helping to denoise individual observations.

### Clustering independent patient groups and simulating reduced observation intervals

To facilitate the translation of our clustering scheme to other cohorts and to understand the degree of generalizability of our clustering solution, we analyzed two aspects:

First, we projected the clustering coefficients to an independent MARS subsample and found that these patients were assigned to classes with similarly shaped group plots and median HAM-D courses as observed for the discovery sample. The observation that the classes formed from the MARS validation sample were also equally proportioned as in the discovery sample confirmed that, within the MARS cohort, a stable solution had been gained. The additional projection onto the GENDEP sample was also informative: Here, patients could be captured equally well by the seven TRCs except for a small proportion of patients that exceeded the lower boundary of one (discovery) cluster due to volatile courses between week 4 and ~10. More relevant, however, significantly different cluster proportions emerged compared with MARS. We speculate that the limited options to intensify treatment in the GENDEP study with defined treatments—or generally different patient characteristics—could underlie the proportional shift towards clusters that represent a slower treatment response. The combination these two observations led us to conclude that indeed generalizable response patterns seem to be described by the seven TRCs. Though, different cluster stability criteria may lead to different solutions, as for example pointed out by a longitudinal latent class analysis that used Bayesian Information Criterion and detected nine clusters in GENDEP^27^. Comparability with our solution, though, is hampered by the use of a different depression rating scale (Montgomery-Åsberg Depression Rating Scale).

Second, in a simulation, we reduced the observation interval to probe whether studies with shorter observation windows could also benefit from the current clustering solution. We found that a correlation of *r* ≈ 0.96 was reached after eight weeks of HAM-D measurements in the MARS-based samples and *r* ≈ 0.77 in the independent GENDEP sample (Fig. 2). Of note, the remaining increase of prediction accuracy between weeks 8 and 12 was stronger in GENDEP, indicating that observation windows of 8 weeks generally seem sufficient, but that, expectedly, differences in study characteristics play a role, rendering more observations advisable. One such difference that could explain the difference at week 8 might have been the higher flexibility in the MARS study to adjust the treatment to the individual patient. Overall, the generalizability of our clustering solution could be higher for observational than for controlled studies.

### Prediction of TRCs from clinical baseline features

We next investigated the clinical usefulness of the TRCs by testing whether these can be predicted from clinical baseline characteristics in a multivariate model (random forest algorithm)^40^. Rather than as a separate study we conceptualized this analysis as additional clinical validation of the clusters that primarily represent statistical constructs. Several machine learning techniques have before been used to predict treatment outcome in MDD^48–50^, yet, their models were directed towards classical categories of remission, non-remission^10^, treatment resistance^12^, or persistence-severity^13^. In brief, we found that 50 clinical baseline variables, obtained through interviews, symptom self-reports, and standard physical or laboratory tests, predicted about 13% of the variance of the TRCs. While seemingly low, this is actually in the range of previous multivariate analyses that focused on the prediction of two outcome categories, reporting low to medium accuracy values from receiver operating characteristic analyses^10,12,13^. In contrary to using pre-defined cutoff thresholds for these categories, clustering as exemplified here for the HAM-D measure can reveal more fine-grained, yet still sparse and data-driven classification systems. Of our clinical predictors, nine carried significantly more weight than the others: (i) the duration of the index episode, (ii–iv) symptom checklist-based scores for psychosocial self-assuredness, psychoticism, and phobic anxiety, (v–viii) the personality traits neuroticism, extraversion, psychoticism, and harm avoidance, and, (ix), sum scores for life events (weighted for their straining impact). Although all items support the overall prediction, a review of these nine items strengthened the clinical validity in several ways:

A longer duration of time in depression before initiation of antidepressant treatment has before been identified as a negative predictor of treatment outcome^51^. In contrary, no consistent predictive value was found for the total duration of the current episode including periods with and without treatment^52,53^. As the period without treatment was not quantified in our sample, we speculate that our current episode duration marker incorporated the untreated period, and significance was gained through the large statistical power. Furthermore, baseline symptom profiles made a relevant contribution to the model. Several reports emphasized that strong anxiety symptoms during a depressive episode increase the risk for non-remission^54^. Of the predictive symptom items (phobic anxiety, psychosocial self-assuredness, and psychoticism) at least two reflect aspects of anxiety, corroborating that high anxiety levels in MDD impede treatment response. Of note, in an analysis on a MARS subsample, patients with high anxiety levels showed structural brain differences in areas involved in the processing of social cues^55^, critically overlapping with areas that predict treatment response over six weeks ^56^.

While the symptom checklist covers state-related items, personality questionnaires target more stable characteristics of a person. Here, harm avoidance and neuroticism—which both represent similar concepts of developing feelings of anxiety and avoidance behavior in the face of challenges—were confirmed as predictors. Such an association has been reported before^57,58^, which constitutes an indirect validation of the TRCs. Extraversion has so far mainly been found to protect against developing clinical symptoms in the face of chronic stress^59^. We report a clearer direct impact on treatment response, a finding possibly facilitated by the random forest approach that integrates multiple interaction effects. Eventually, weighted life events emerged as a negative predictor, as reported^60,61^. Life events, particularly early adverse events, represent episodes of prolonged adaptation, stress, and liability that increase the risk for MDD, but that also influence recovery chances^51–53,62,63^. Information on early childhood adversity was only available in a subsample (≈35%), disqualifying it for the full model. We speculate that the inclusion of additional details on the type and timing of life events could improve the model.

In an earlier representative MARS sample^29^, previous treatment resistance—usually defined by at least two unsuccessful trials with different antidepressants in adequate dosages for at least six weeks^64^—has been identified as a strong univariate predictor of non-remission. In this study, treatment resistance was encoded by the Antidepressant Treatment Response Questionnaire (ATRQ) that showed no significant importance *p*-value (yet a significant univariate association [data not shown]). Results based on the ATRQ may differ because this measure tends to underreport failed trials^65^. Similarly, the BMI, previously reported to be associated with remission rates^29^ and treatment response^66^, was not associated with the TRCs in our study. One explanation is the use of a binary cutoff (25 kg/m^2^) in the positive report^66^, which may point to a non-linear relationship. Of note, the number of previous depressive episodes—a lifetime disease burden marker—did not emerge as a predictor, confirming other negative reports^64^. Similarly, age at onset (AAO), which is often inversely correlated with the number of episodes, was not predictive. Concerning this marker, reports are mixed, some finding no correlation^67,68^ and some reporting an influence on remission speed^69^ or treatment resistance^70^. Hidden interactions of AAO with subgroups (as reported for comorbid alcohol dependency)^71^ or non-linear relationships may explain this variability. Baseline cortisol as a simple HPA axis marker was also not predictive; stimulation tests, particularly when obtained longitudinally, are most likely more sensitive^72^. TRCs also differed by the type of psychopharmacological treatment (Supplementary Table S2), yet, due to the observational study design, this likely reflects either disease acuity (anxiolytic medication), treatment escalation following non-response (e.g., tricyclic antidepressants), or episode severity (antipsychotic medication for psychotic depression). Similar confounding co-correlations between medication variables and disease severity have been reported for biological markers, e.g., in meta-analyses of brain structure ^73,74^.

We explored two different strategies for improving our base model 0 (Table 1), by either adding single baseline HAM-D items or by adding information on the partial early response after 2 weeks. Interestingly, the inclusion of single baseline HAM-D items did not improve the model (Table 2), possibly because the current symptomatology was already reflected in the symptom checklist items. This does not imply that *primary* clustering of single item trajectories would not result in additional clusters. While representing an important follow-up question and adding clinical elaborateness, this conceptual modification would increase the number of observations per case and could lead to model instability. Eventually, including the partial early response increased the model fit markedly, confirming similar reports from both observational and controlled studies^12,16,48–50,62,63^. Notably, personality items were among the strongest predictors in all models (Table 3).

### Limitations

Our study has several limitations. First, due to a necessary tradeoff between higher statistical power through a large sample size and the use of powerful, specific single predictors, clinical variables like neurocognitive results, complex endocrine tests, or neuroimaging markers were not included, despite reports on them being potentially useful^72,75^. Second, while psychopharmacological treatments are well-documented in MARS, no formalized assessment of previous non-pharmacological treatments, including psychotherapy, was available, preventing an inclusion of these factors. Third, the MARS discovery and validation samples significantly differed in six clinical baseline items, which may explain minor differences of the prediction results. However, these six items showed no overlap with the most informative predictors of model 0 or predictors emerging from the other models.

## Conclusions

By employing model-based non-linear clustering to clinical ratings of a large cohort of MDD patients, we detected seven distinct treatment response classes that proved stable in two validation samples. In a multivariate prediction analysis, these classes could be predicted from 50 clinical baseline variables, with personality items, life events, duration of the episode, and psychopathological baseline characteristics carrying particular weight. Overall, the construct and clinical validity of these treatment response classes in MDD encourages an exploration of their neurobiological underpinnings and, more generally, effectively describes response patterns across multiple clinical cohorts.

## Supplementary information

Supplemental Data File

Supplemental Figure 1

Supplemental Figure 2
