# Supplementary material for: Treatment response classes in major depressive disorder identified by model-based clustering and validated by clinical prediction models
Source: Transl Psychiatry. 2019 Aug 5;9:187. doi: 10.1038/s41398-019-0524-4 (PMC6683145; doi:10.1038/s41398-019-0524-4)
Supplement: Supplementary file 1 — Supplemental Data File [file 41398_2019_524_MOESM1_ESM.docx]

**Supplementary Material**

**Supplemental Figure S1.** **Specification of four clinical sets of predictors for the prediction model.** *Model 0* (see methods and **Table 1** for detailed specification of all clinical items), and three extended models (*model 1, model 2* and *model 3*). Random forest prediction models were estimated for all four sets of predictors with each two variants of modelling the treatment response slope (individual slope vs. cluster-derived slope).

**
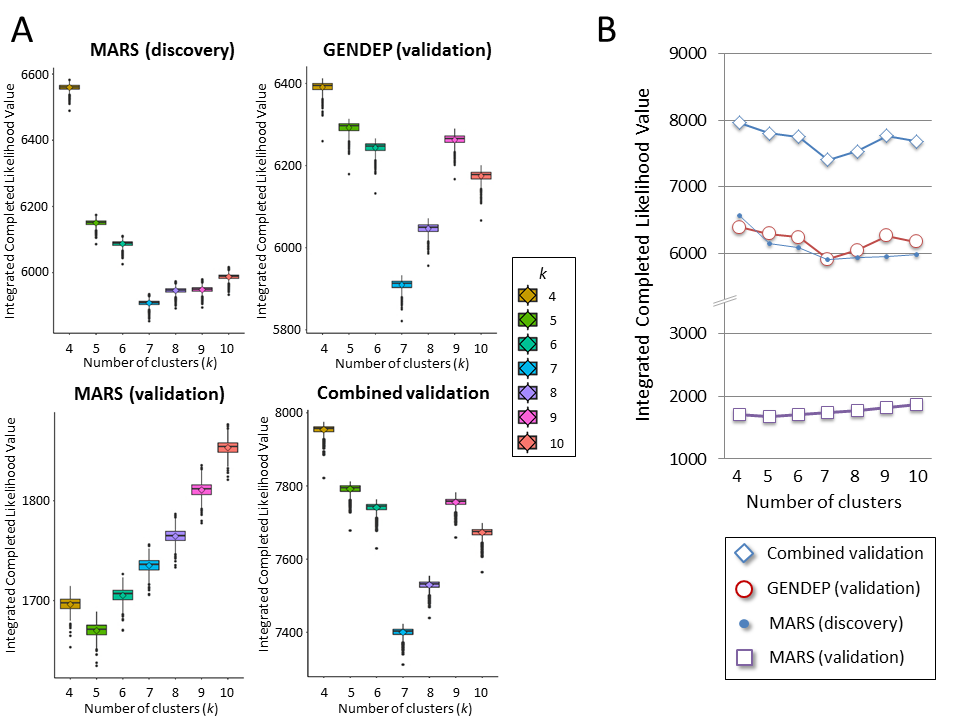
**

**Supplemental Figure S2. Integrated completed likelihood (ICL) values of the discovery sample and the validation samples**. *(A)* ICL values for clustering solutions between 4 and 10 clusters are plotted on scales adjusted to the respective result range. One data point represents one subject; open diamonds represent mean values; vertical lines represent one standard error of the mean; boxplots represent the median and quartiles. *(B)* For improved comparability, ICL mean values of the MARS discovery sample, the MARS validation sample, the GENDEP validation sample and the combined validation samples (MARS and GENDEP) are plotted on the same Y-axis.

**Supplemental Table S1. Description of demographic items for all different type of samples**

|  | Variable description | Short name | Type | MARS discovery sample | | MARS validation  sample | | GENDEP validation  sample | |
| --- | --- | --- | --- | --- | --- | --- | --- | --- | --- |
|  |  |  |  | Mean | SD | Mean | SD | Mean | SD |
|  |  |  |  | % | | % | | % | |
|  | Age at study inclusion (years) | age | *N* | 48.26 | 14.02 | 45.48 | 14.99 | 41.69 | 11.58 |
|  | Sex (% female) | sex | *D* | 53.72 % | | 53.39 % | | 64.10% | |
|  | Living with a partner | spouse | *D* | 50.24% | | 38.56% | | 57.35% | |
|  | School years of education (university not considered) (years) | education | *N* | 10.33 | 1.46 | 10.21 | 1.51 | 10.94 | 1.28 |
|  | Being in training/retirement *vs.* employment  (% employment) | training_ retirement | *D* | 25.42% | | 22.46% | | 18.28% | |
|  | Employment status: unemployed/part time/full time | employment | *N* | 1.54 | 0.78 | 1.56 | 0.78 | 1.28 | 0.90 |

**Supplemental Table S2. Percentage of imputed clinical items**

| Category | Short name | Proportion of imputed values | Category  (cont’d) | Short name  (cont’d) | Proportion of imputed values  (cont’d) |
| --- | --- | --- | --- | --- | --- |
| Socio­demo­graphic data | age | 0% | Life events | L-Event | 41.49% |
|  | sex | 0% |  | wL-Event | 41.86% |
|  | spouse | 2.61% | Baseline psycho­pathology | scl_som | 19.53% |
|  | education | 5.23% |  | scl_comp | 19.71% |
|  | training_ retirement | 3.73% |  | scl_uncert | 19.62% |
|  | employment | 28.5% |  | scl_dep | 19.43% |
| Diagnosis | ICD10 | 0% |  | scl_anx | 19.62% |
| History of depressive disorder | age_on | 4.39% |  | scl_agg | 20.09% |
|  | prev_epi | 16.44% |  | scl_pho | 19.71% |
|  | s_history | 12.14% |  | scl_par | 19.81% |
|  | psychot_history | 4.20% |  | scl_psy | 20.09% |
| Family history | fam_history | 2.80% | Personality items | epq_neu | 37.47% |
|  | fam_F20_F25 | 1.78% |  | epq_psy | 37.57% |
|  | fam_F31 | 1.78% |  | epq_ext | 37.66% |
|  | fam_F32_ _F34 | 1.59% |  | tpq_ha | 37.38% |
|  | fam_X60 | 1.78% |  | tpq_ns | 37.38% |
| Information on current episode | index_d | 10.0% |  | tpq_rd | 37.47% |
|  | ATRQ_Score | 21.96% |  | tpq_rd2 | 37.29% |
|  | s_current | 9.81% |  |  |  |
|  | psychot_current | 0% |  |  |  |
| Basic medical and baseline  laboratory data | height | 2.89% |  |  |  |
|  | weight | 7.66% |  |  |  |
|  | BMI | 8.13% |  |  |  |
|  | HR | 2.61% |  |  |  |
|  | RRsys | 2.61% |  |  |  |
|  | RRdia | 2.52% |  |  |  |
|  | cort_basal | 62.99% |  |  |  |
|  | TSH | 12.89% |  |  |  |
|  | fT3 | 35.42% |  |  |  |
|  | fT4 | 35.14% |  |  |  |
|  | CRP | 54.29% |  |  |  |
|  | HbA1C | 64.76% |  |  |  |

**Supplemental Table S3. Baseline HAM-D and average HAM-D values per cluster (discovery sample)**

| **Cluster label** | **Baseline HAM-D**  **[mean (SD)]** | **Average**  **HAM-D across time series**^b^ **[mean (SD)]** | **Comparison of neighboring clusters** | | |
| --- | --- | --- | --- | --- | --- |
|  |  |  | **Cluster pair** | **Baseline HAM-D**  **[mean (SD)]** | **Average**  **HAM-D across time series**^a^ **[mean (SD)]** |
| C1^a^ | 24.63 (6.08) | 11.15 (3.74) | C1 *vs.* C2 | 0.006 | 2.10×10^-9^ |
| C2 | 26.35 (6.26) | 14.15 (3.74) | C2 *vs.* C3 | n. s.^d^ | 0.018 |
| C3 | 26.10 (6.14) | 15.47 (3.95) | C3 *vs.* C4 | n. s. | 0.001 |
| C4 | 26.22 (6.65) | 12.46 (3.53) | C4 *vs.* C5 | n. s. | 7.11×10^-12^ |
| C5 | 26.71 (4.86) | 19.17 (4.07) | C5 *vs.* C6 | n. s. | 0.005 |
| C6 | 25.10 (6.44) | 16.23 (4.30) | C6 *vs.* C7 | n. s. | 4.25×10^-8^ |
| C7 | 27.04 (6.03) | 21.80 (4.56) |  |  |  |
| ANOVA (df=6) | *p*=9.446×10^-3^ | *p*=4.022×10^-116^ |  |  |  |
| Linear correlation^c^ | *r*=0.09, *p*=2.497×10^-3^ | *r*=0.57, *p*=8.271×10^-76^ |  |  |  |

^a^ Sorting is by increasing cluster-derived slope, as in **Figure 1**.

^b^ Average across all available HAM-D values of the time series until discharge.

^c^ Correlation between cluster-derived slope and individual baseline HAM-D values (middle column) and individual average HAM-D across time (right column).

^d^ n. s., not significant (*p*>0.05)

**Supplemental Table S4. Association between TRCs, established response markers and psycho­pharmacological treatment in the combined MARS sample**

|  | **Clinical item** | **ANOVA/**  *Χ^2^* **test**^a^  ***p*-value** | **Cohen’s *f*** | **Cohen’s ω** |
| --- | --- | --- | --- | --- |
| **Established response markers** | Response (HAM-D reduction >50%) at discharge | 4.16×10^-65^ | N/A | 0.546 |
|  | Remission (HAM-D<10) at discharge | 1.05×10^-90^ | N/A | 0.609 |
|  | Weeks until discharge | 4.59×10^-84^ | 0.685 | N/A |
| **Psycho-pharmacological treatment**^b^ | Tricyclic antidepressants | 1.75×10^-5^* | 0.177 | N/A |
|  | Selective serotonin reuptake inhibitors | 0.071 | 0.104 | N/A |
|  | Selective serotonin and noradrenalin reuptake inhibitors | 0.477 | 0.077 | N/A |
|  | Noradrenergic and specific serotonergic antidepressants | 0.503 | 0.063 | N/A |
|  | Other antidepressants | 0.012 | 0.126 | N/A |
|  | Antipsychotic medication | 9.68×10^-12^* | 0.247 | N/A |
|  | Mood stabilizers | 0.012 | 0.130 | N/A |
|  | Anxiolytic medication | 1.44×10^-7^* | 0.197 | N/A |
|  | Sleep promoting medication | 0.229 | 0.090 | N/A |

^a^ *Χ^2^* test for categorical variables response and remission. ANOVA was applied if not otherwise specified.

^b^ Pharmacological treatment classes were binary coded (1 := applied/0 := not applied) every week and then aggregated across the entire hospitalization period, with “1” indicating treatment with the respective drug category during complete hospitalization, “0” indicating not applied at all, and values between “0” and “1” indicating the relative time under treatment with the respective drug category. Most patients were treated with several types of pharmacological treatments.

**p*-value robust towards Bonferroni correction for nine psycho-pharmacological classes.

**Supplemental Table S5. Comparison of established response markers between neighboring clusters in the MARS discovery sample**

|  | **Cluster averages** | | | **Comparison of neighboring clusters** | | | |
| --- | --- | --- | --- | --- | --- | --- | --- |
| **Cluster** | **Response**  (>50% HAM-D reduction at discharge) | **Remission**  (HAM-D<10 at discharge) | **Weeks until discharge** | **Cluster pair** | **Response**  (>50% HAM-D reduction at discharge) | **Remission**  (HAM-D<10 at discharge) | **Weeks until discharge** |
| C1 | 100.0% | 100.0% | 4.9 | C1 *vs.* C2 | n.s.^a^ | 7.38×10^-6^ | 6.05×10^-13^ |
| C2 | 85.8% | 98.7% | 6.7 | C2 *vs.* C3 | 1.78×10^-5^ | 2.59×10^-4^ | 4.99×10^-18^ |
| C3 | 76.1% | 91.6% | 11.3 | C3 *vs.* C4 | n. s. | 9.13×10^-3^ | 0.002 |
| C4 | 88.2% | 91.2% | 14.0 | C4 *vs.* C5 | 8.75×10^-6^ | 1.58×10^-16^ | n. s. |
| C5 | 25.0% | 57.5% | 12.4 | C5 *vs.* C6 | n. s. | 1.16×10^-5^ | 4.19×10^-4^ |
| C6 | 59.3% | 80.7% | 16.8 | C6 *vs.* C7 | 1.87×10^-8^ | 1.22×10^-9^ | 0.003 |
| C7 | 12.9% | 32.5% | 20.8 |  | | | |

^a^ n. s., not significant (*p*>0.05)

**Supplemental Table S6. Overview of classification accuracy (%) per class for all models**

| **Model** | **Sample** | **C1**^a^ | **C2** | **C3** | **C4** | **C5** | **C6** | **C7** |
| --- | --- | --- | --- | --- | --- | --- | --- | --- |
| **Model 0** | All | 84.4 | 80.4 | 78.3 | 95.1 | 81.5 | 91.7 | 88.2 |
|  | Discovery | 83.0 | 79.7 | 78.9 | 95.2 | 81,8 | 92.0 | 88.6 |
|  | Replication | 89.0 | 82.2 | 75.4 | 94.5 | 79.7 | 90.7 | 86.9 |
| **Model 1** | All | 84.6 | 80.4 | 78.3 | 95.1 | 81.4 | 91.7 | 88.4 |
|  | Discovery | 83.2 | 79.7 | 78.8 | 95.2 | 81.9 | 92.0 | 88.8 |
|  | Replication | 88.6 | 82.6 | 75.0 | 94.5 | 79.7 | 90.7 | 86.9 |
| **Model 2** | All | 84.7 | 80.4 | 78.3 | 95.1 | 81.5 | 91.7 | 88.5 |
|  | Discovery | 83.2 | 79.7 | 78.9 | 95.2 | 81.8 | 92.0 | 88.7 |
|  | Replication | 89.0 | 82.2 | 75.0 | 94.5 | 79.7 | 90.7 | 86.9 |
| **Model 3** | All | 84.6 | 80.4 | 78.3 | 95.1 | 81.4 | 91.7 | 88.5 |
|  | Discovery | 83.8 | 79.7 | 78.8 | 95.2 | 81.9 | 92.0 | 89.0 |
|  | Replication | 88.6 | 82.6 | 75.4 | 94.5 | 80.1 | 90.7 | 86.9 |

^a^ Classification accuracy defined as [true positives + true negatives]/[true positives + false positives + true negatives + false negatives])

**Supplemental Table S7. Demographic variables compared across clusters and samples**

| **Demographic**  **variables** | **Differences between clusters (*p-*value)** | | | **MARS discovery & validation** | **All 3 samples** |
| --- | --- | --- | --- | --- | --- |
|  | **MARS discovery** | **MARS**  **validation** | **GENDEP** |  |  |
| Age^a^ | 0.135 | 0.251 | 3.463×10^-05^ | 0.150 (C)^c^  0.006 (S)^d^  0.223 (C×S)^e^ | 0.008 (C)  1.268×10^-21^ (S)  0.048 (C×S) |
| Sexr^b^ | 0.276 | 0.298 | 0.005 | 0.999 (C)  0.981 (S)  0.975 (C×S) | 0.999 (C)  0.002 (S)  0.247 (C×S) |
| Spouse^b^ | 0.222 | 0.684 | 0.890 | 0.999 (C)  0.007 (S)  0.999 (C×S) | 0.999 (C)  1.270×10^-5^ (S)  0.984 (C×S) |
| Education^b^ | 0.098 | 0.071 | 0.127 | 0.999 (C)  0.958 (S)  0.999 (C×S) | 0.999 (C)  1.571×10^-21^ (S)  0.837 (C×S) |
| Employment^b^ | 0.404 | 0.788 | 0.062 | 0.999 (C)  0.986 (S)  0.999 (C×S) | 0.999 (C)  1.390×10^-5^ (S)  0.903 (C×S) |
| Training/  Retirement^b^ | 0.365 | 0.022 | 0.915 | 0.999 (C)  0.635 (S)  0.775 (C×S) | 0.999 (C)  0.015 (S)  0.460 (C×S) |

^a^ ANOVA used for continuous variables.

^b^ Chi square statistics for categorical values.

^c^ (C) stands for *p*-values for main effect of cluster.

^d^ (S) stands for *p*-values for main effect of sample.

^e^ (C×S) stands for *p*-values for the cluster-by-sample effect.

**Supplemental Table S8. Overview of significant predictor variables of all models (combined MARS sample)**

| **Category** | **Clinical item**^a^ | **Model 0**  *p*-value^bc^ | **Model 1**  *p*-value | **Model 2**  *p*-value | **Model 3**  *p*-value |
| --- | --- | --- | --- | --- | --- |
| **Personality items** | epq_neu | <0.0001 | <0.0001 | <0.0001 | <0.0001 |
|  | epq_psy | 0.0444 | 0.0433 | 0.0192 | 0.0308 |
|  | epq_ext | <0.0001 | <0.0001 | <0.0001 | <0.0001 |
|  | tpq_ha | <0.0001 | <0.0001 | <0.0001 | <0.0001 |
| **Life events** | L-Event | n.s | 0.0220 | n. s. | n. s. |
|  | wL-Event | 0.0002 | <0.0001 | 0.0008 | 0.0002 |
| **Baseline psycho-pathology** | scl_comp | n. s. | 0.0304 | n. s. | n. s. |
|  | scl_uncert | 0.0073 | 0.0020 | 0.0215 | 0.0099 |
|  | scl_pho | 0.0207 | 0.0156 | n.s | 0.0327 |
|  | scl_psy | 0.0182 | 0.0155 | n. s. | 0.0308 |
|  | HAM-D0_13^d^ | not  included | 0.0327 | not  included | 0.0045 |
|  | HAM-D0_14^d^ |  | 0.0258 |  | n. s. |
|  | HAM-D0_16^d^ |  | 0.0419 |  | n.s |
| **Information on current episode** | index_d | 0.0210 | 0.0186 | n. s. | n. s. |
|  | psychot_ current | n. s. | n. s. | 0.0466 | 0.0492 |
| **Early response rated at  week 2** | HD_2WE | not  included | not  included | <0.0001 | <0.0001 |

^a^ epq_neu: neuroticism (EPQ-RK); epq_psy: psychoticism (EPQ-RK); epq_ext: extraversion (EPQ-RK); tpq_ha: harm avoidance total (TPQ); L-Event: sum of life events; wL-Event: sum of weighted life events; scl_comp: compulsiveness (SCL-90R); scl_uncert: uncertainty in social contact (SCL-90R); scl_pho: phobic anxiety (SCL-90R); scl_psy: psychotism (SCL-90R); index_d: duration of current episode; psychot_current: Psychotic symptoms during the current episode; HD_2WE: HAM-D early partial response (≥25% reduction) after 2 weeks. Categories/items that delivered no predictors at *p*<0.05 are not listed in the table.

^b^ *p*-values are based on testing the respective single importance value against all other competing predictors (see methods for details).

^c^ *p*-values for model 0 are identical with the *p*-values reported in **Table 2** and listed for direct comparison with the other models.

^d^ baseline item HAM-D items: 13: somatic symptoms – general, 14: genital symptoms, 16: weight loss

*Abbreviations:* n. s., not significant (p>0.05)
