# Supplementary figures and images for: Treatment response classes in major depressive disorder identified by model-based clustering and validated by clinical prediction models
Source: Transl Psychiatry. 2019 Aug 5;9:187. doi: 10.1038/s41398-019-0524-4 (PMC6683145; doi:10.1038/s41398-019-0524-4)

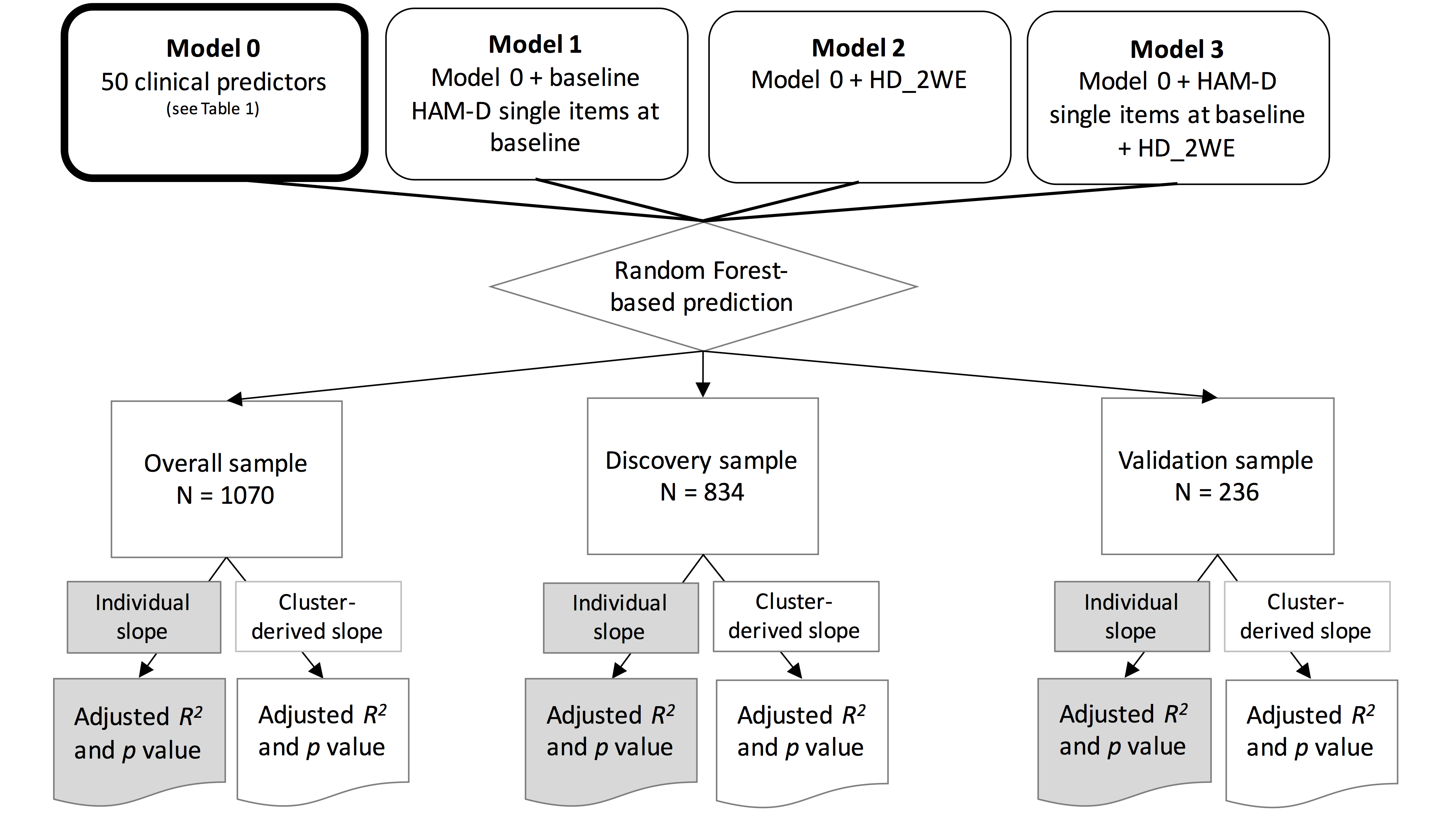

Supplement: Supplementary file 2 — Supplemental Figure 1 [file 41398_2019_524_MOESM2_ESM.tif]

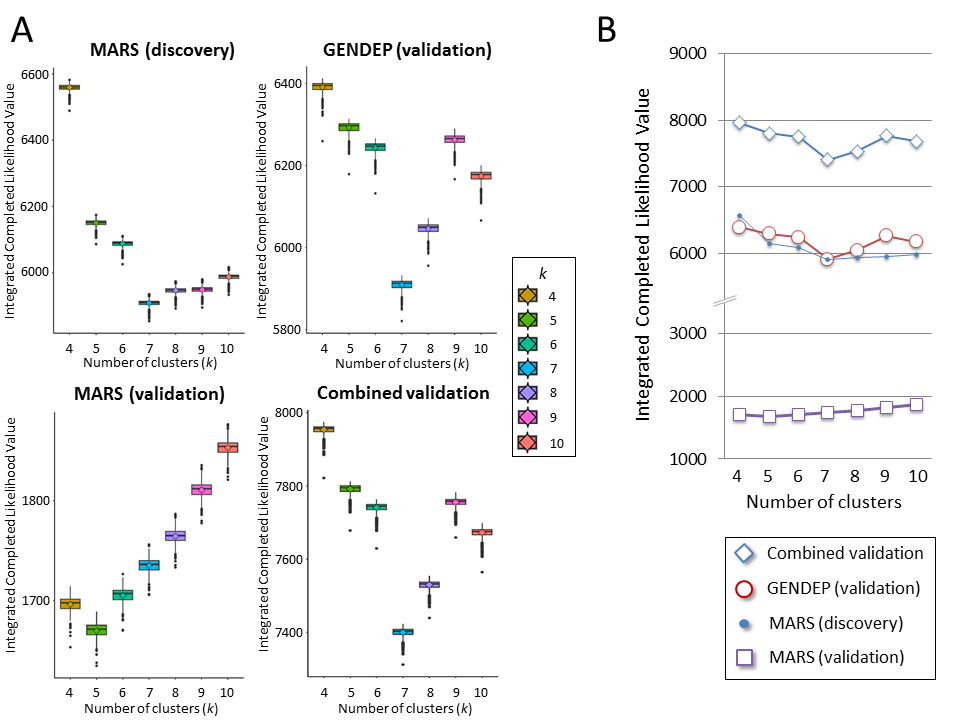

Supplement: Supplementary file 3 — Supplemental Figure 2 [file 41398_2019_524_MOESM3_ESM.png]
